# Indoor Localization with Extended Trajectory Map Construction and Attention Mechanisms in 5G

**DOI:** 10.3390/s25185784

**Published:** 2025-09-17

**Authors:** Kexin Yang, Chao Yu, Saibin Yao, Zhenwei Jiang, Kun Zhao

**Affiliations:** 1 The Engineering Center of SHMEC for Space Information and GNSS, East China Normal University, Shanghai 200241, China; 51265904009@stu.ecnu.edu.cn (K.Y.); cyu@sist.ecnu.edu.cn (C.Y.); jiangzhenwei1@chinaunicom.cn (Z.J.); 2 Shanghai Branch of China Unicom, Shanghai 200082, China; yaosaibin1@chinaunicom.cn

**Keywords:** 5G, fingerprint positioning, indoor localization, generative adversarial network

## Abstract

Integrated sensing and communication (ISAC) is considered a key enabler for the future Internet of Things (IoT), as it enables wireless networks to simultaneously support high-capacity data transmission and precise environmental sensing. Indoor localization, as a representative sensing service in ISAC, has attracted considerable research attention. Nevertheless, its performance is largely constrained by the quality and granularity of the collected data. In this work, we propose an attention-based framework for cost-efficient indoor fingerprint localization that exploits extended trajectory map construction through a novel trajectory-based data augmentation (TDA) method. In particular, fingerprints at unmeasured locations are synthesized using a conditional Wasserstein generative adversarial network (CWGAN). A path generation algorithm is employed to produce diverse trajectories and construct the extended trajectory map. Based on this map, a multi-head attention model with direction-constrained auxiliary loss is then applied for accurate mobile device localization. Experiments in a real 5G indoor environment demonstrate the system’s effectiveness, achieving an average localization error of 1.09 m and at least 34% higher accuracy than existing approaches.

## 1. Introduction

Integrated sensing and communication (ISAC) has shown great potential for intelligent networks and the Internet of Things (IoT). It is widely recognized as a key driver for the evolution of future wireless networks [1]. Numerous innovative applications, including autonomous mobility [2], virtual/augmented reality [3], and location-based services (LBSs) [4], require close collaboration between wireless communication and environmental sensing in beyond-fifth generation (B5G) and sixth-generation (6G) networks [5,6]. As a typical application of ISAC, indoor localization through wireless signals plays a crucial role across various scenarios. In most cases, wireless signals are reflected or absorbed, making satellite-assisted technologies insufficient for accurate indoor positioning [7,8]. As a result, indoor localization has become an increasingly critical area of research.

As the fifth generation of mobile networks, 5G provides significant advantages for indoor localization, including better energy efficiency, low latency, and the ability to support massive device connectivity. Among the various reference signals in 5G networks, the synchronization signal–reference signal received power (SS-RSRP) captures essential indoor radio propagation characteristics and is widely accessible on commercial devices without extra hardware cost [9,10], making it a more practical feature compared to positioning reference signals (PRSs) and channel state information (CSI). Motivated by these advantages, this study focuses on indoor localization methods based on SS-RSRP.

**Figure 1:**
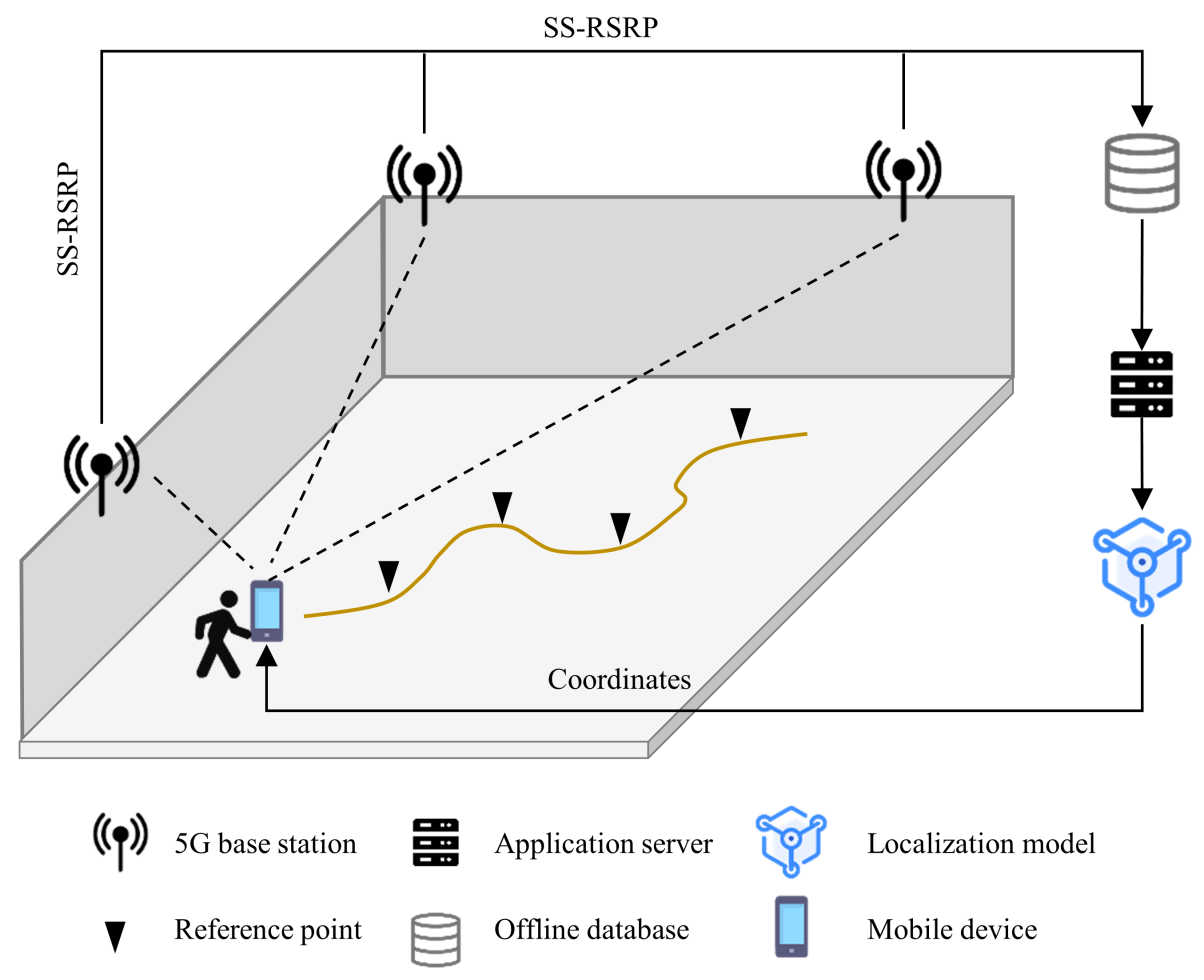
Schematic of indoor positioning for a mobile device.

A fingerprint-based localization system typically operates in two phases: offline and online. As illustrated in Figure 1, in the offline phase, the area of interest is partitioned into reference points (RPs), where received signal strength (RSS) measurements from available base stations (BSs) are collected to construct an offline database. In the online phase, RSS measurements of a mobile device (MD) from unknown locations are fed into a localization model trained on a server to estimate the position [11,12]. Owing to their robustness and adaptability in complex and dynamic indoor environments, fingerprinting-based approaches have come to be among the most widely adopted solutions for indoor positioning.

Fingerprint-based localization methods can be broadly categorized into traditional machine learning (ML) algorithms and deep learning (DL) approaches. Classical ML algorithms, such as k-nearest neighbors (KNN) [13], support vector machines (SVM) [14], and random forests (RF) [15], have long been employed for indoor localization but are primarily designed for static positioning. In contrast, DL methods leverage the powerful feature extraction capabilities of neural networks, typically achieving higher localization accuracy than ML approaches and gaining considerable traction in trajectory prediction tasks. For example, some studies have utilized convolutional neural networks (CNNs) [16,17] to enable accurate feature extraction. Long short-term memory (LSTM) networks [18,19,20] are also applied to achieve higher accuracy at the cost of increased computational complexity. Recently, transformer architectures have gradually been introduced into wireless signal processing and localization due to their powerful global modeling capabilities and efficient handling of sequential data [21,22,23]. Accordingly, this study concentrates on DL-based fingerprint localization.

However, indoor localization of mobile devices in ISAC still faces two major challenges: (1) construction of the detailed map and (2) the dynamic prediction of device positions during movement. The details of these challenges and the proposed solutions are outlined as follows.

Challenge 1: The positioning accuracy of fingerprinting algorithms strongly depends on the size and coverage of the dataset. Collecting wireless signal measurements through on-site surveys is both time-consuming and labor-intensive, and achieving full coverage of the localization area is often impractical. Existing data augmentation methods mainly focus on static point enhancement, with limited consideration for the diversity enhancement of dynamic trajectories.

Challenge 2: In indoor scenarios involving device mobility, location estimation becomes inherently dynamic, with variations in movement states and instability in received signals substantially degrading the accuracy of trajectory prediction. Traditional fingerprinting-based localization systems, which rely on single signal measurements, are particularly vulnerable to wireless interference and often suffer from large deviations in positioning results.

Motivated by the above challenges, we propose a trajectory-based localization framework that combines data augmentation with a multi-head attention model. Our trajectory-based data augmentation (TDA) algorithm employs a generative adversarial network (GAN) to synthesize fingerprints at unmeasured locations, while a path generation procedure links synthetic and real points to form continuous trajectories. This expands the trajectory map without additional data collection, effectively enriching fingerprint diversity for improved localization. By constructing an extended trajectory map, the generated data can be effectively applied to trajectory prediction, overcoming limitations in prior studies. The augmented dataset is then exploited by a multi-head attention model to capture spatio-temporal dependencies in dynamic SS-RSRP sequences. Furthermore, an auxiliary loss with directional constraints is introduced to refine trajectory prediction, ensuring closer alignment with the ground truth. The proposed method enables efficient construction of the trajectory map with low cost overhead and fully leverages trajectory information to enhance the accuracy of trajectory prediction.

The main contributions of this study are summarized as follows.

(1) Trajectory-based data augmentation: In TDA, synthetic fingerprints at unmeasured locations are generated using a conditional Wasserstein generative adversarial network (CWGAN). An artificial path generation algorithm then links real and synthetic points to create diverse trajectories and construct an extended trajectory map, thereby improving both the density and variability of the training dataset.
(2) Attention-based localization model with an auxiliary loss: We employ a multi-head attention mechanism to learn spatial–temporal dependencies and dynamic variations in collected SS-RSRP sequences. To reduce trajectory prediction error, an auxiliary loss based on directional constraints is incorporated during model training, resulting in predicted trajectories that more closely match the ground truth.
(3) Validation of performance: Extensive real-world experiments in a 5G system with device mobility are conducted to evaluate the performance. The results show that our method significantly outperforms existing localization methods.

The remainder of this paper is organized as follows. Section 2 reviews related work. Section 3 introduces the system architecture and details the proposed framework. Section 4 describes the experimental setup and discusses the results. Finally, Section 5 concludes the paper.

## 2. Related Work

In this section, we provide a brief overview of the related studies on fingerprinting-based localization and data augmentation in extended radio map construction.

### 2.1. Fingerprinting Approaches

Fingerprinting-based localization algorithms can be classified into two categories: probabilistic approaches and deterministic approaches. Probabilistic methods [24,25] model the uncertainty in signal measurements and try to estimate the most probable location by calculating the likelihood between the real-time samples and those stored in the pre-established database. Deterministic approaches estimate the user’s location by computing the similarity between real-time and offline fingerprints, relying on ML and DL algorithms to infer positions. Traditional ML methods, such as KNN [13], SVM [14], and RF [15], are mainly limited to static indoor localization scenarios. With the advancement of artificial intelligence (AI), DL models have been increasingly adopted to enhance localization accuracy and generalization. CNNs [26,27] transform wireless signals collected at reference points (RPs) into fingerprint images to extract latent spatial features. This reformulates the localization task as an image classification or regression problem. To effectively capture the temporal correlations in fingerprint sequences, the authors of [18] proposed LSTMs to leverage the temporal correlation of RSSI measurements. In recent years, attention networks have gained popularity in indoor localization, as they are effective at learning long-range relationships and handling time-series signal data. The studies reported in [22,23] utilized attention mechanisms to process RSS matrices for high-accuracy positioning. In [28], a hybrid CNN–Transformer model is proposed for localization in LoRaWAN networks, where CNNs extract local features and the Transformer captures global dependencies.

Although these DL-based methods outperform traditional ML approaches, they still require large amounts of data to train the model for high localization accuracy. The labor-intensive data collection process limits practicality and scalability.

### 2.2. Data Augmentation Methods

To reduce the overhead of data collection, traditional methods like kriging interpolation (KI) [29] and GPR [30] generate additional samples within the original dataset. However, these approaches suffer from limited extrapolation capability, high computational complexity, and sensitivity to hyperparameter selection. Recently, GANs have been leveraged to synthesize new fingerprint data due to their strong generative abilities. The method proposed in [31] converts collected CSI data into amplitude feature maps and extends the fingerprint database using a convolutional generative adversarial network. The authors of [32] proposed a generation network that augments CSI by integrating location features with randomly sampled spatial features from the target domain to preserve consistency in dynamic environments. However, the acquisition of CSI requires additional hardware costs. The authors of [33] presented a GAN-based approach for the generation of additional point-based training data at unmeasured locations. Despite its effectiveness in enriching static fingerprints, the method does not address the diversity of trajectories, which is essential for supporting localization during device movement. MapLoc [34] employs Gaussian process regression for data augmentation at unmeasured locations to expand radio maps. However, its strategy of training models on the augmented map and fine tuning on the original map limits robustness in predicting unmeasured locations and increases the complexity of model training. These augmentation methods fail to construct an extended trajectory map, making them unsuitable for accurate localization of moving devices.

A comparison of RSS-based related works is summarized in Table 1. The limitations in trajectory diversity enhancement and dynamic localization motivate our work.

## 3. System Modeling

### 3.1. System Overview

**Figure 2:**
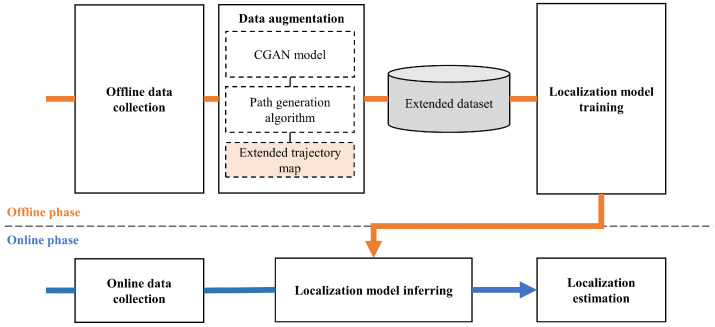
System overview.

Figure 2 illustrates the workflow of the proposed method, which consists of an offline phase and an online phase. In the offline phase, a SLAM-equipped robot collects fingerprints with 2D coordinates. To reduce the cost of constructing a training dataset and to boost localization accuracy, data augmentation is proposed to generate fingerprints at unmeasured locations, followed by the building of an extended trajectory map. A multi-head attention model with auxiliary loss is then trained on the extended dataset to capture latent dependencies in SS-RSRP sequences and enhance robustness to measurement instability. In the online phase, SS-RSRP sequences collected from moving MDs are fed into the well-trained localization model for location prediction. The following subsections provide a detailed description.

### 3.2. Data Preparation

**Figure 3:**
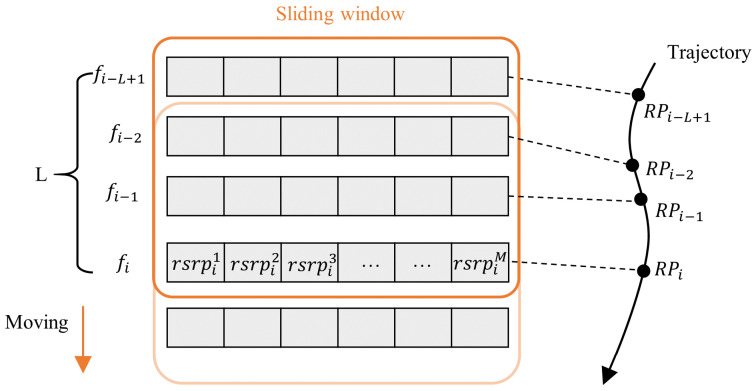
Serialization of SS-RSRP readings via a sliding window.

We utilize a robot equipped with 16-line LiDAR and a smartphone (Huawei P40) to perform automated data collection along predefined routes. As shown in Figure 3, the collected signal data along one path is segmented into sequences using a sliding window of length *L*. At time $t_{i}$, the fingerprint at the *i*-th RP is represented as $f_{i}=\left(rsrp_{i}^{1},rsrp_{i}^{2},\ldots ,rsrp_{i}^{M}\right)$, with its corresponding coordinate denoted as $p_{i}=\left(x_{i},y_{i}\right)$, where $rsrp_{i}^{j}$ is the SS-RSRP value received from the *j*-th BS at the *i*-th RP. *M* denotes the total number of 5G BSs. At time $t_{i}$, the fingerprint sequence ($s_{i}$) contains the fingerprints from the current and the previous L−1 steps:

(1) $$s_{i}=\left(f_{i-L+1},f_{i-L+2},\ldots ,f_{i}\right)$$

The coordinate ($p_{i}$) of the RP at time $t_{i}$ serves as the label of $s_{i}$. The sliding window then moves forward with a stride of 1 to obtain the next fingerprint sequence, and this process continues iteratively over the entire path. Due to factors such as long distances from BSs, signal interruptions, and physical obstructions, some SS-RSRP measurements may be unavailable. To maintain input consistency, missing SS-RSRP values are replaced with the minimum detectable threshold of −157 dBm, and the data are subsequently normalized using min–max scaling.

### 3.3. Trajectory-Based Data Augmentation (TDA)

**Figure 4:**
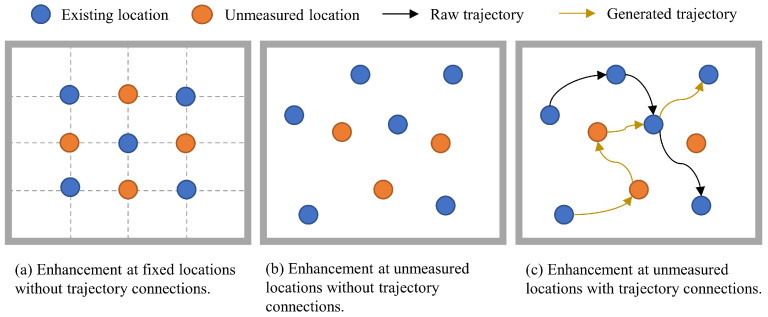
Illustration of data augmentation strategies: (**a**) fixed point-based augmentation; (**b**) random point-based augmentation; (**c**) trajectory-based data augmentation.

Indoor localization is fundamentally limited by the scarcity of high-quality datasets. Existing augmentation methods primarily generate additional fingerprints at fixed or random unmeasured locations but lack path continuity, making them unsuitable for dynamic trajectory prediction, where temporal correlations are essential. Figure 4 presents a comparison of various data augmentation methods applied in indoor localization.

To overcome these challenges, we propose TDA, a framework for constructing an extended trajectory map. TDA not only generates fingerprints at unmeasured locations to increase dataset granularity but also synthesizes diverse trajectories by linking generated and original data. The framework comprises two key steps:

(1) Synthetic fingerprint generation at unmeasured locations using CWGAN;
(2) Extended trajectory map construction using a path generation algorithm.

#### 3.3.1. Generating Synthetic Fingerprints Using CWGAN

**Figure 5:**
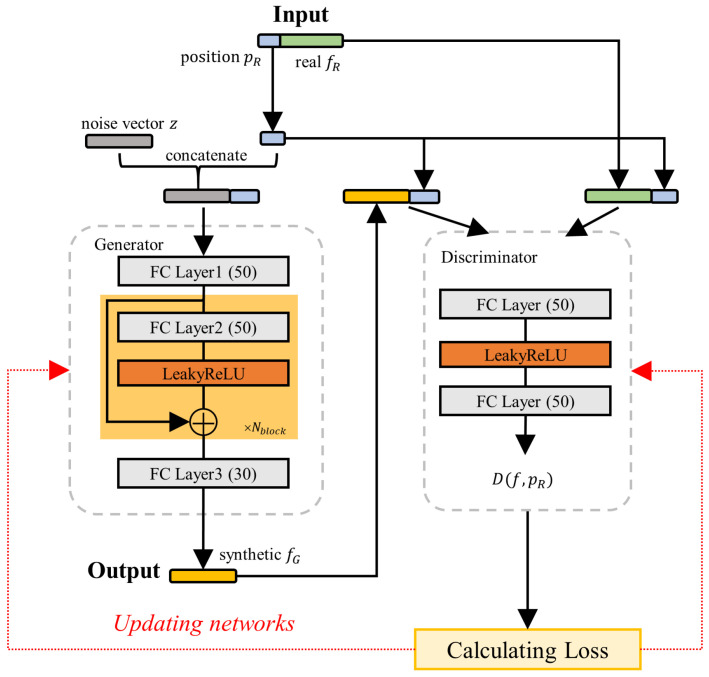
Conditional WGAN framework.

GANs have been widely applied to data augmentation in domains such as image and speech processing. A traditional GAN [36] consists of a generator and a discriminator. The generator aims to produce synthetic data samples that closely resemble real data, while the discriminator distinguishes between real and generated samples. These two components compete against each other until the generator produces data nearly indistinguishable from real samples, making it difficult for the discriminator to differentiate between them. To address unstable training and mode collapse in GANs, WGAN [37] introduces a gradient penalty to enforce the 1-Lipschitz constraint, thereby ensuring more stable training. Inspired by WGAN, we adopt a conditional WGAN in our framework, as illustrated in Figure 5.

Given real fingerprints ($f_{R}$) and corresponding coordinates ($p_{R}$) as conditions, the input of the generator consists of a vector formed by concatenating $p_{R}$ with a set of random noise vectors ($z∼P_{z}$, where $P_{z}$ denotes the standard normal distribution). The generator (*G*) comprises a sequence of fully connected (FC) layers designed to capture the underlying distribution of fingerprint features. The input first passes through FC layers to perform feature transformation. It then flows through $N_{block}$ residual blocks, each consisting of FC layers and LeakyReLU activation, to further model the distribution of SS-RSRP. Residual connections are added to each block to facilitate gradient flow during training and to mitigate the degradation problem in deep networks. Then, the intermediate representation is mapped to an M-dimensional feature vector. The output of the generator is synthetic fingerprints ($f_{G}=G\left(z,p_{R}\right)$). The input fingerprints (*f*) of discriminator *D* originate from either the real fingerprints ($f_{R}$) or the synthetic fingerprints ($f_{G}$). The output of the discriminator is a score ($D(f,p_{R})$), which represents the similarity between $f_{G}$ and $f_{R}$. The loss functions are defined as follows:

(2) $$L_{G}=-E_{f∼P_{g}}\left[D\left(f,p_{R}\right)\right]$$

(3) $$\begin{array}{c} L_{D}=E_{f∼P_{g}}\left[D\left(f,p_{R}\right)\right]-E_{f∼P_{r}}\left[D\left(f,p_{R}\right)\right]+\lambda E_{\hat{f}∼P_{\hat{f}}}\left[{\left({∥\nabla_{\hat{f}}D\left(\hat{f},p_{R}\right)∥}_{2}-1\right)}^{2}\right] \end{array}$$

where $P_{r}$ represents the real data distribution, $P_{g}$ denotes the synthetic data distribution, and $P_{\hat{f}}$ refers to the distribution of samples interpolated between points sampled from $P_{r}$ and $P_{g}$. The term λ is a tuning factor that adjusts the gradient penalty. To ensure that the generator sufficiently captures the underlying distribution of the input features, we train the generator $n_{critic}$ times for each update of the discriminator. During training, the Adam optimizer is used. The parameters of the CWGAN are summarized in Table 2.

After training, a set of unmeasured locations ($p_{G}$) is randomly sampled within the localization area. These locations are then employed as generation conditions for the synthesis of new fingerprints ($f_{G}=G\left(z,p_{G}\right)$).

#### 3.3.2. Extended Trajectory Map Construction

**Figure 6:**
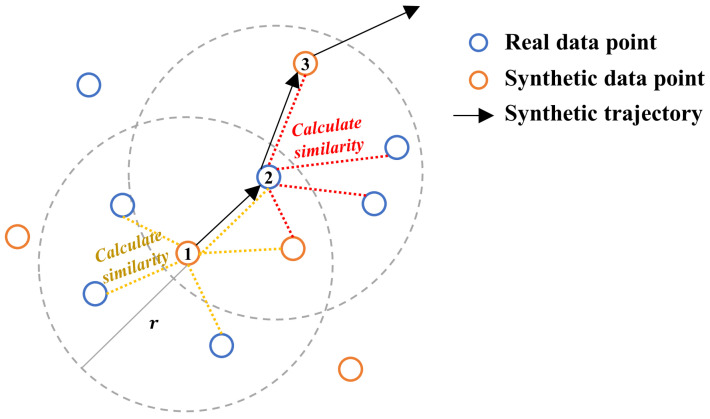
The process of the synthetic path generation algorithm. The numbers represent the sequential indices of the points.

As mentioned earlier, the goal of TDA is to construct an extended trajectory map. In open areas, MDs’ moving directions and step lengths tend to be more variable. To enhance the diversity of trajectories, we design a directionally constrained path generation algorithm to produce new trajectories, as described in Algorithm 1. The process begins by randomly selecting two adjacent RPs from the original and synthetic points, labeled 1 and 2. Assuming that an MD rarely makes long-distance jumps between adjacent positions, the location of the next point is generally close to that of the current point. As illustrated in Figure 6, the next point is selected from candidate points located within a fixed radius of *r* = 1 m centered at the current location.
**Algorithm 1:** Artificial path generation algorithm.
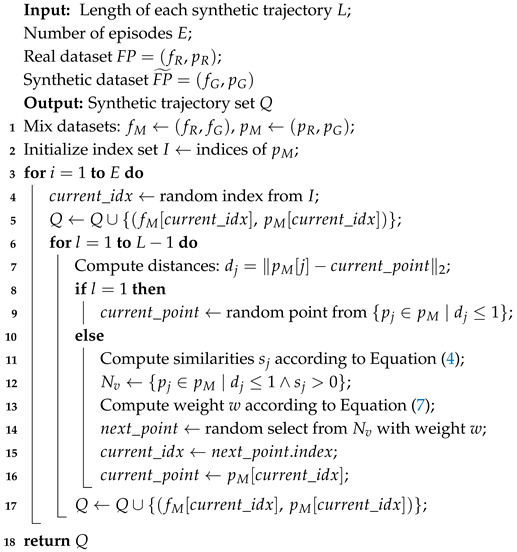


Since MDs typically do not change direction drastically within a short time, we propose selecting the next point from candidate points based on directional similarity. Candidate points may originate from both real and synthetically generated locations. Given the current point ($p_{t}$) and the previous point ($p_{t-1}$), the directional similarity between $p_{t}$ and its *i*-th candidate ($p_{t,i}$) is defined as follows:

(4) $$\begin{array}{cc} s(t,i) & =\frac{v_{i}\cdot d_{t}}{∥v_{i}∥\cdot ∥d_{t}∥} \end{array}$$

(5) $$\begin{array}{cc} v_{i} & =p_{t,i}-p_{t} \end{array}$$

(6) $$\begin{array}{cc} d_{i} & =p_{t}-p_{t-1} \end{array}$$

The similarity score (s(t,i)) increases as the candidate point’s direction more closely aligns with the MD’s current movement direction. To ensure smooth trajectories, directional changes between consecutive steps are limited to within 90 degrees, thereby restricting valid candidates to those with s(t,i)>0. The next point is selected based on the similarity weights of these valid candidates:

(7) $$w_{t,i}=\frac{s(t,i)}{\sum_{j=1}^{n}s\left(t,j\right)}$$

where *n* represents the total number of valid candidates. The procedure is iteratively performed to generate a set of synthetic trajectories, each of a length of *L*. In this way, the extended trajectory map is constructed by mixing the original and generated trajectories.

### 3.4. Localization Model

**Figure 7:**
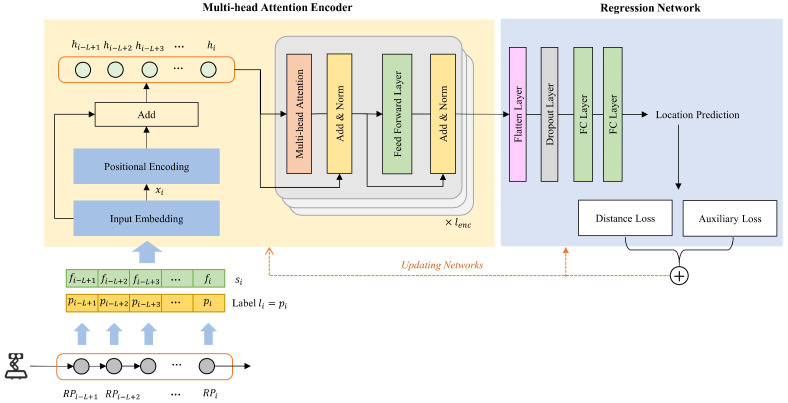
The architecture of the proposed localization model.

To extract dynamic features from sequences of SS-RSRP measurements across consecutive steps, we propose a localization model that integrates an encoder with a multi-head attention mechanism, inspired by the Transformer architecture proposed in [38]. As illustrated in Figure 7, the overall architecture consists of a multi-head attention encoder and a regression network. We model the spatial–temporal relationships between any positions in a sequence by leveraging this multi-head attention encoder, achieving a superior ability to capture latent dependencies compared to RNNs and CNNs. Unlike the canonical Transformer, our model omits the decoder and instead adopts a regression network for position estimation.

The position of one RP at the final time step is assigned as the label such that $l_{i}=p_{i}$. The SS-RSRP sequence ($s_{i}=\left(f_{i-L+1},f_{i-L+2},\ldots ,f_{i}\right)$) is first projected into a $d_{model}$-dimensional space via an input embedding layer:

(8) $$x=s_{i}W_{e}+b_{e},x\in R^{L\times d_{model}}$$

where $W_{e}\in R^{M\times d_{model}}$ and $b_{e}\in R^{d_{model}}$ are learnable parameters. The input embedding is capable of learning and encoding the relationships among SS-RSRP values from different BSs. Meanwhile, positional encoding is employed to incorporate order information into the input sequence, which is formulated as follows:

(9) $$\begin{array}{cc} PE_{\left(pos,2j\right)} & =\sin\left(\frac{pos}{10000^{2j/d_{\mathrm{model}}}}\right) \end{array}$$

(10) $$\begin{array}{cc} PE_{\left(pos,2j+1\right)} & =\cos\left(\frac{pos}{10000^{2j/d_{\mathrm{model}}}}\right) \end{array}$$

where pos denotes the position of an element in the sequence ($x_{i}$) and *j* is the dimensional index. The outputs of positional encoding and input embedding are added to form the latent representation. The encoder comprises $l_{enc}$ stacked encoding blocks. The multi-head self-attention mechanism in each block is formally defined as follows:

(11) $$\begin{array}{cc} Multihead(Q,K,V) & =Concat\left(H_{1},H_{2},\ldots ,H_{h}\right)W^{0} \end{array}$$

(12) $$\begin{array}{cc} Attention(Q,K,V) & =softmax(\frac{QK^{T}}{\sqrt{d_{k}}})V \end{array}$$

(13) $$\begin{array}{cc} H_{i} & =Attention(QW_{i}^{Q},KW_{i}^{K},VW_{i}^{V}) \end{array}$$

where *K*, *Q*, and *V* denote the key, query, and value matrices derived from previous latent representations. $W_{i}^{Q}\in R^{d_{model}\times d_{k}}$, $W_{i}^{K}\in R^{d_{model}\times d_{k}}$, $W_{i}^{V}\in R^{d_{model}\times d_{v}}$, and $W^{0}\in R^{h_{head}d_{v}\times d_{model}}$ are weight matrices. $d_{k}$, $d_{v}$, and $d_{model}$ represent the dimensions of corresponding vectors and the encoder layers. *h* is the number of attention heads. Finally, we employ a regression network to directly project the encoder output to a 2D geographic space. The regressor comprises flattenedand fully connected layers with LeakyReLU activation functions.

In addition, an auxiliary loss function with directional constraints is introduced to model training. This design encourages the predicted coordinates to closely match the ground truth in both distance and direction, thereby mitigating abrupt directional changes, which usually do not match the movement patterns of the mobile device. The overall loss function comprises two components: a distance error loss ($L_{d}$) and a direction variation loss ($L_{a}$). Specifically, $L_{d}$ is formulated as the mean squared error (MSE) between the predicted and actual coordinates:

(14) $$L_{d}=MSE\left(\hat{p},p\right)$$

where $\hat{p}$ denotes the predicted coordinate and *p* represents the corresponding ground-truth coordinate. $L_{a}$ is a direction-constrained auxiliary loss that improves continuous location prediction by penalizing deviations between the predicted and true movement directions at each time step, thereby mitigating abrupt trajectory changes. The moving directions of the predicted and true trajectories at time *t* are defined as follow:

(15) $$\begin{array}{c} v_{pred}={\hat{p}}_{t}-p_{t-1} \end{array}$$

(16) $$\begin{array}{c} v_{true}=p_{t}-p_{t-1} \end{array}$$

The angle between these two directions is determined as follows:

(17) $$cos\theta =\frac{v_{pred}}{∥v_{pred}∥+\varepsilon }\cdot \frac{v_{true}}{∥v_{true}∥+\varepsilon }$$

Here, ε is a small constant ($1\times 10^{-6}$) introduced to prevent division by zero. Then, $L_{a}$ is calculated to penalize cases where the angle exceeds a certain threshold ($\theta_{thr}$):

(18) $$L_{a}=\max\left(|\theta |-\theta_{thr},0\right)$$

Finally, the overall loss function is defined as the weighted sum of $L_{a}$ and $L_{d}$:

(19) $$Loss=L_{d}+\alpha L_{a}$$

where α is a weighting factor that controls the relative contribution of $L_{a}$. The model employs the Adam optimizer with a learning rate of 0.001. Dropout and an early stopping strategy are applied to reduce overfitting. Detailed parameter settings of the proposed localization model are listed in Table 3.

### 3.5. Performance Metrics

To evaluate the performance of the proposed system, two commonly used metrics are adopted: the mean absolute error (MAE) and its cumulative distribution function (CDF). Additionally, following the 3GPP [39] requirements on positioning accuracy, the errors corresponding to the 80% and 90% cumulative probabilities are observed for evaluation. The MAE between the predicted coordinates ($\hat{p}=\left(\hat{x},\hat{y}\right)$) and the true position (p=(x,y)) is defined as follows:

(20) $$MAE\left(\hat{p},p\right)=\frac{1}{N_{p}}\sum_{r=1}^{N_{p}}{\left[{\left({\hat{x}}_{r}-x_{r}\right)}^{2}+{\left({\hat{y}}_{r}-y_{r}\right)}^{2}\right]}^{\frac{1}{2}}$$

where $N_{p}$ is the total number of testing samples.

## 4. Experiment and Performance Analysis

### 4.1. Experimental Setup

**Figure 8:**
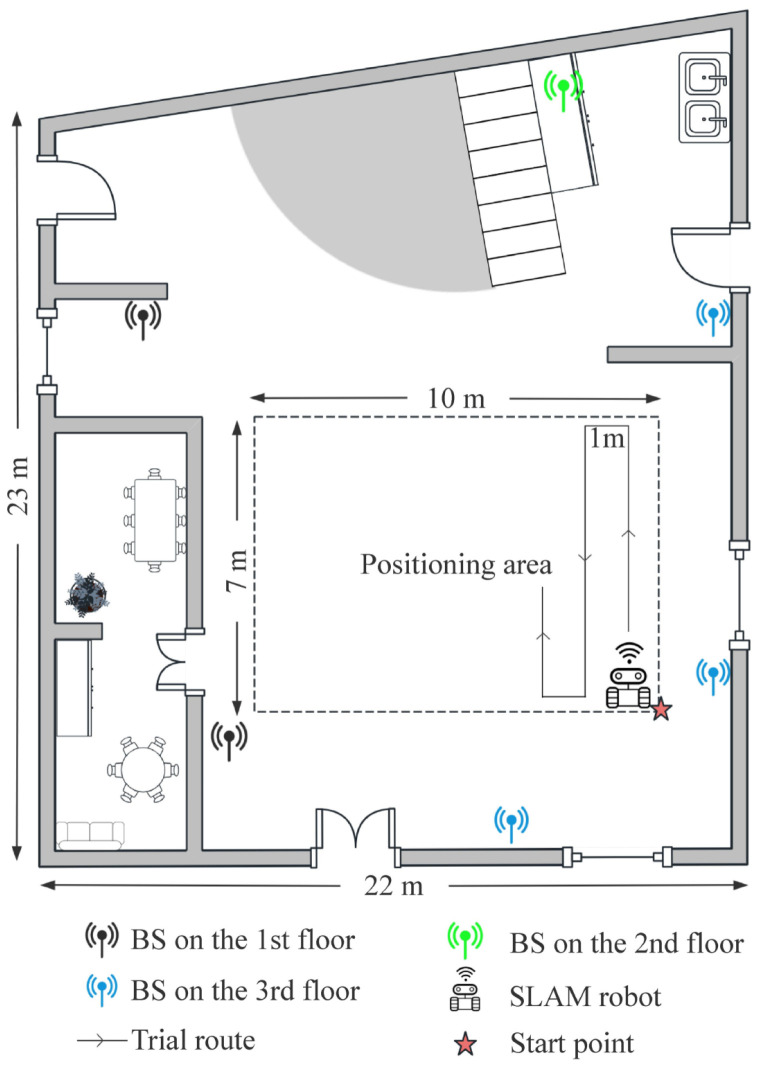
Indoor localization environment and base station deployment.

To evaluate the effectiveness of the proposed localization approach, we conducted experiments in a hall on the first floor of an academic building. The hall covers an area of approximately 500 $m^{2}$. As illustrated in Figure 8, six 5G BSs are deployed across different floors: two on the first floor, one on the second floor, and three on the third floor. For clarity, BSs on different floors are marked in different colors in the figure. This multi-floor deployment introduces significant challenges for signal propagation, as wireless signals are subject to attenuation and obstruction by the building’s structure. Due to the presence of walls between multiple floors and the height differences in base station deployment, the positioning scenario can be regarded as a non-line-of-sight (NLOS) environment, which poses significant challenges to achieving accurate localization. To maintain a clear space for regular human activity, a 10 × 7 m area at the center of the hall was designated as the positioning area. During data collection, the environment contained moving pedestrians, contributing to a realistic and dynamic testing scenario.

**Figure 9:**
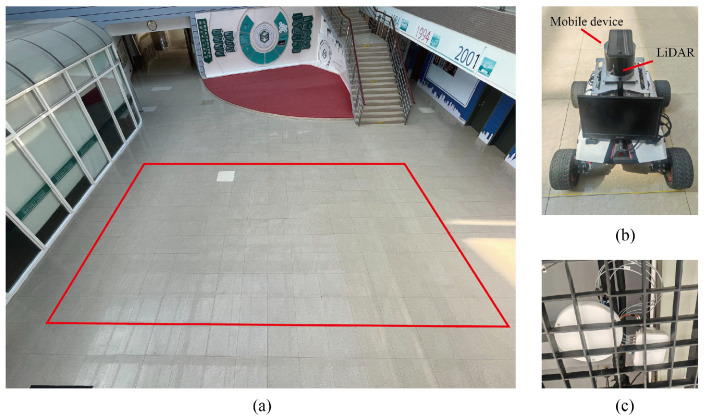
Experimental setup. (**a**) The spatial layout of the localization area. (**b**) The device with a light detection and ranging (LiDAR) sensor and a mobile phone for data collection. (**c**) Base stations.

During the offline training phase, data were collected using a mobile robot equipped with a 16-line light detection and ranging (LiDAR) sensor and a Huawei P40 smartphone mounted on its top, as shown in Figure 9. The robot autonomously navigated predefined trajectories within the designated area. During movement, the smartphone continuously captured SS-RSRP from surrounding 5G BSs, while the robot simultaneously recorded its 2D coordinates using SLAM-based localization. The robot maintained a constant speed of 1 m/s to simulate pedestrian movement and recorded both position and signal measurements at 1 s intervals. At each time step, one sample comprises the SS-RSRP readings from all BSs and the corresponding 2D location. All SS-RSRP values were preprocessed by missing-value imputation and min–max normalization. The data were then segmented into sequences using a sliding window with a length of *L*. In total, 2096 sequences were collected and split into training and validation sets at a ratio of 8:2.

During the online testing phase, the mobile device was rebooted and reconnected to BSs. Unlike the training phase, where movement followed a predetermined trajectory, the robot moved randomly throughout the entire localization area to construct the test dataset. This approach aims to closely simulate real-world positioning scenarios, where the target MD does not always follow straight trajectories. The collected test data were also preprocessed through missing-value imputation and normalization. Consecutive samples were segmented into multiple sequences of length *L*, which were then fed into the pretrained localization model to output predicted coordinates. The test set comprised 300 sequences, ensuring no overlap with the training set. Detailed experimental settings are summarized in Table 4.

All experiments were performed on a computer with a 13th Gen Intel Core i5-13400 processor (2.50 GHz) and 16 GB of RAM, running Windows 10. The software environment included Python 3.8 and PyTorch 2.3.0.

### 4.2. Accuracy of Location Estimation

Compared with existing methods, our approach expands the trajectory map without additional data collection cost and achieves more accurate and robust trajectory prediction using a multi-head attention mechanism and a direction-constrained auxiliary loss. To evaluate the performance of our method, we compare it with traditional ML algorithms and leading DL methods. ML algorithms include KNN [13], SVM [14], and RF [15]. In addition, several leading DL methods are compared, including those that exploit sequential inputs for trajectory localization (e.g., CNN/RNN architectures [26,27,34] and attention-based models [22,23]), as well as localization approaches enhanced through data augmentation [34,40]. For a fair comparison, all methods used the same test points. The localization results of each method are summarized and compared in Table 5. For all baseline methods, the relative percentage improvement in MAE achieved by the proposed approach was evaluated. The results indicate that our proposed model achieves improved localization accuracy, with average positioning errors reduced by at least 47% and 34% compared to ML and DL methods, respectively. In the following, we discuss these results in detail.

**Figure 10:**
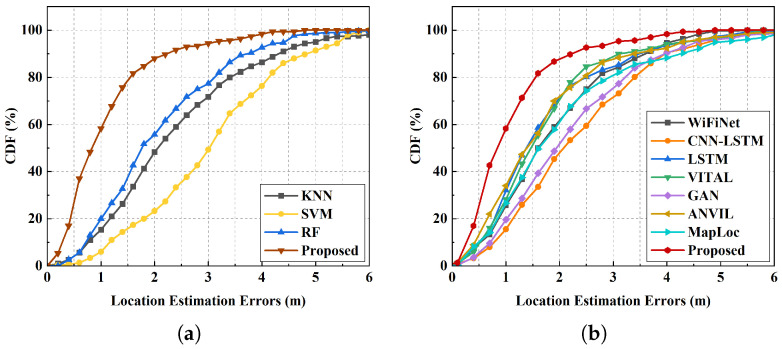
Comparison of localization errors for (**a**) ML methods and (**b**) DL methods.

In general, DL approaches outperform traditional ML methods in localization accuracy. Traditional ML algorithms typically generate predictions based on individual samples, making them more vulnerable to signal fluctuations and environmental noise. As shown in Figure 10, ML methods yield average localization errors in the range of 2 m to 3 m. In contrast, DL approaches deliver superior localization performance, benefiting from their greater capacity for hierarchical feature extraction and representation learning.

To assess the benefits of trajectory-based localization, we first compare the proposed method with point-based DL approaches, such as CNN-LSTM and GAN. CNN-LSTM captures inter-feature dependencies but ignores temporal information, while GAN-based methods augment data at unmeasured points yet still perform single-point localization. Environmental dynamics, such as pedestrian activity, may lead to distribution shifts between training and deployment data, thereby degrading localization accuracy. Our model leverages multi-head attention to model global relationships across multiple steps rather than relying solely on instantaneous measurements. Our method outperforms these methods by over 49% under sequence data consideration to capture spatio-temporal correlations and mitigate NLOS and multipath effects, achieving lower errors, with an MAE under 2 m, as shown in Table 5.

**Figure 11:**
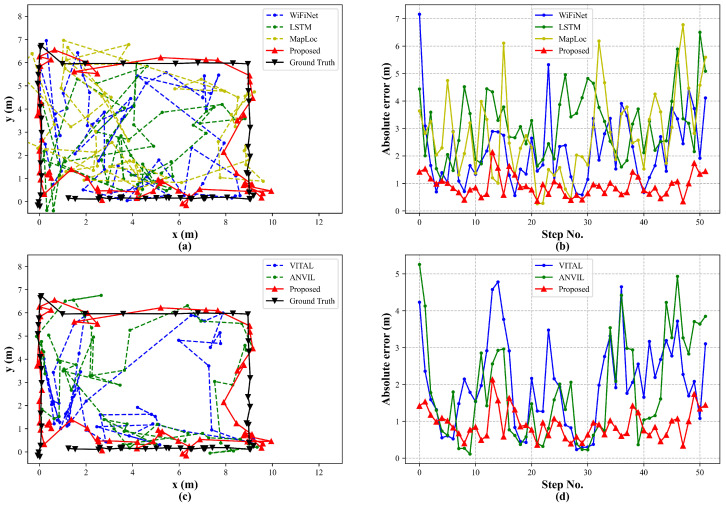
Comparison of trajectory prediction results using different sequence-based methods: (**a**) CNN/LSTM-based predicted trajectories; (**b**) CNN/LSTM-based step-wise localization errors; (**c**) attention-based predicted trajectories; (**d**) attention-based step-wise localization errors.

To further validate the effectiveness of the proposed attention-based localization model, we compared it with other trajectory prediction methods. A test set was collected along a trajectory near the boundary of the localization region. Figure 11 presents both the predicted trajectories and the step-wise localization errors. The results show that our method yields predictions much closer to the ground truth and consistently outperforms other sequence-based approaches. WiFiNet mainly captures local features through convolution, limiting its ability to model dynamic trajectory changes. LSTMs improve sequential prediction but suffer from step-by-step dependency of wireless signals, which reduces robustness in complex environments. In contrast, the attention mechanism captures global temporal dependencies, making our model more resilient in predicting dynamic movement.

As for data augmentation, TDA is introduced for the construction of an extended trajectory map. For trajectory prediction, diverse trajectories are required, rather than isolated point data, to enhance the model’s feature extraction and fitting capability. VITAL and ANVIL apply simple image-domain augmentations, such as adding Gaussian noise or adjustments to brightness and contrast at existing locations. Although GAN generates new fingerprints at unmeasured locations to enrich the dataset, it still lacks path continuity. These approaches provide limited trajectory diversity. In addition, unlike MapLoc, which trains and fine tunes models separately on the generated and original datasets, our approach constructs an extended trajectory map on a mixed dataset. This enables simultaneous learning of fingerprint features from both existing and unmeasured locations, thereby reducing the risk of overfitting to a single type of dataset. Our method achieves an MAE of 1.09 m, which is 34.73%, 35.88%, and 45.50% lower than VITAL, ANVIL, and MapLoc, respectively. The 80% and 90% CDF errors are only 1.50 m and 2.23 m. These results validate the effectiveness of constructing extended trajectory maps via TDA in significantly enhancing trajectory prediction accuracy.

In summary, the proposed method effectively augments the dataset and expands the trajectory map, achieving the lowest localization error by leveraging TDA and the multi-head attention mechanism.

### 4.3. Ablation Study

To evaluate the contribution of each component of the proposed model, we conducted an ablation study to analyze the impact of TDA and the localization network on localization accuracy.

#### 4.3.1. Validity of TDA

The performance of indoor localization for moving MDs is closely influenced by both the number of trajectories and their spatial distribution. To improve localization accuracy, we proposed TDA to construct an extended trajectory map as described in Algorithm 1.

**Figure 12:**
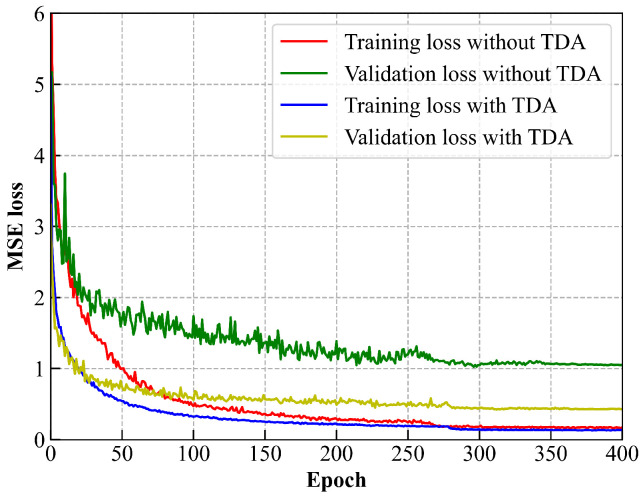
The training and validation loss curves of the localization model.

We compared the localization performance with and without TDA. Based on empirical observations, the number of unmeasured locations is set equal to that of the original RPs. As shown in Figure 12, without TDA, the localization model quickly overfits the training set, yielding a low training loss but consistently high validation loss due to distribution mismatch. With TDA, the model converges faster, and the loss gap between training and validation decreases. These results indicate that TDA improves both data diversity and training efficiency.

**Figure 13:**
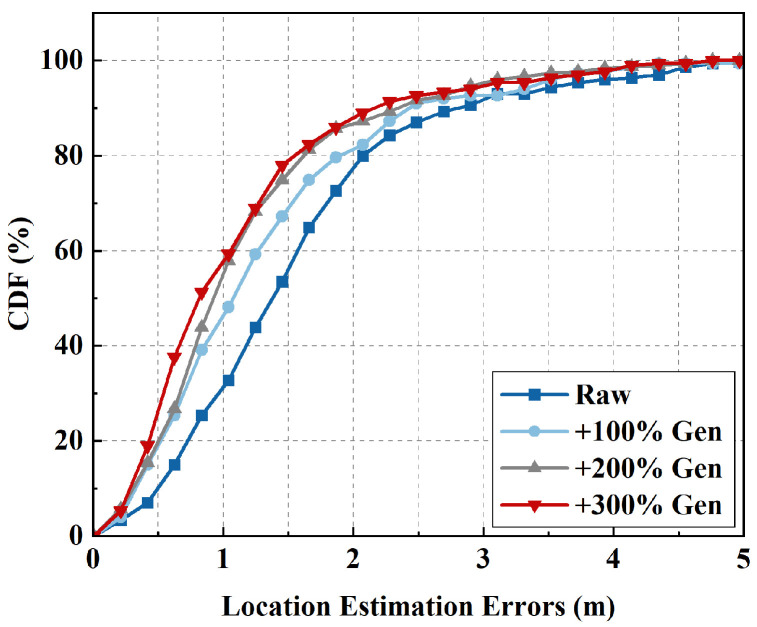
CDFs of localization error under varying numbers of generated samples.

We further evaluated the performance of TDA under varying numbers of generated samples. As shown in Figure 13, “+200% Gen” indicates that the number of generated trajectories is twice that of the original ones. Results show a consistent decrease in localization error with more generated samples, confirming the effectiveness of TDA. Using only the raw dataset achieves an MAE of 1.55 m, with CDF80% and CDF90% errors of 2.09 m and 2.83 m, respectively. With 100% generated samples, these errors decrease to 1.31 m, 1.88 m, and 2.45 m, representing reductions of 15.48%, 10.05%, and 13.43%, respectively. With 300% generated samples, errors drop further to 1.09 m (MAE), 1.50 m (CDF80%), and 2.23 m (CDF90%), achieving an improvement of approximately 30%. However, increasing the generated samples yields diminishing improvements and higher training costs. Therefore, “+300% Gen” offers the best trade-off between accuracy improvement and efficiency. These results further validate that TDA substantially enhances localization accuracy.

#### 4.3.2. Impact of Multi-Head Attention and Auxiliary Loss

**Figure 14:**
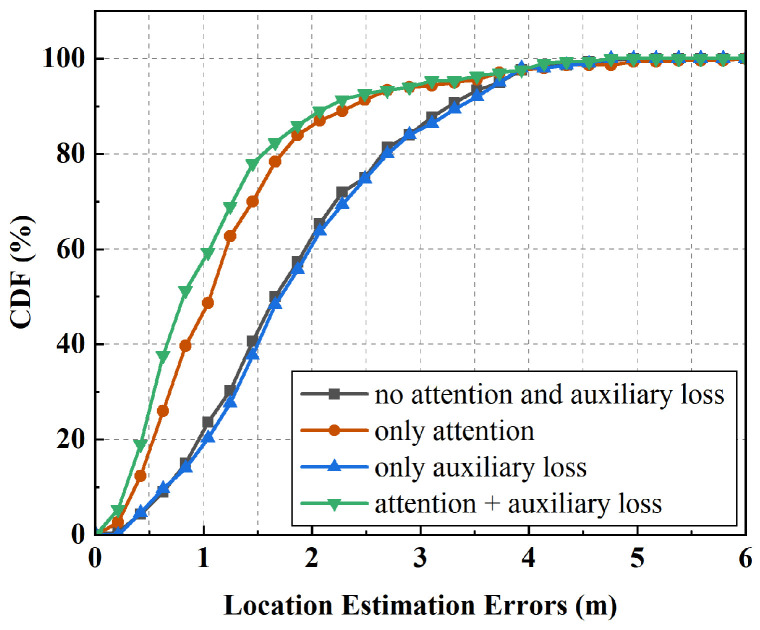
Impact of multi-head attention and auxiliary loss on localization accuracy.

We conducted ablation experiments to evaluate the impact of the multi-head attention mechanism and the auxiliary loss on localization accuracy. By selectively removing the attention mechanism and the auxiliary loss, we assessed their individual contributions to error reduction. As shown in Figure 14, the combination of the two mechanisms achieves the lowest localization error, with an MAE of 1.09 m, representing a reduction of 0.16 m compared to using either the attention mechanism or the auxiliary loss alone and a reduction of 0.74 m relative to using neither. With the constraint of the auxiliary loss, the predicted movement directions between consecutive positions more closely follow the true trajectory, with fewer abrupt turns, thereby reducing overall localization error. Notably, the multi-head attention and the auxiliary loss reduce the localization error by approximately 31.87% and 12.9%, respectively. These findings demonstrate that incorporating the auxiliary loss and attention mechanism significantly contributes to accuracy improvement.

### 4.4. Hyperparameter Analysis

In this section, we conduct experiments to examine the impact of key hyperparameter, including the sequence length, the number of encoder layers, and the number of attention heads. To ensure fair comparison, we focus solely on the parameters of the localization model, excluding data augmentation, which has been discussed in the previous section.

We first compare the average localization error under different sequence lengths. As illustrated in Table 6, the results indicate that shorter sequences are more susceptible to noise from signal fluctuations, while increasing *L* helps smooth out dynamic variations and yields more stable localization results. When *L* = 4, the model achieves the lowest MAE of 1.55 m. However, further increasing *L* leads to a slight degradation in performance. This is because the current position is more strongly influenced by recent movements. Longer sequences may introduce noise due to outdated or irrelevant observations. In addition, longer sequences incur higher computational costs. A sequence length of *L* = 4 (i.e., sliding window size) is found to be optimal.

To discuss the impact of the number of attention heads, we fixed the layers of the multi-head attention encoder and varied the number of attention heads. As shown in Table 7, different head configurations result in minor performance differences. Using four attention heads achieved the lowest MAE values of 1.08 m and 1.64 m on the validation and test sets. We further adjusted the number of encoder layers. The results presented in Table 8 indicate that the model achieved lower localization errors when the encoder consisted of two or three layers. However, the performance gap between these two configurations was negligible on the validation set. Deeper architectures enhanced the model’s feature extraction capability but also increased the risk of overfitting and computational overhead. Considering the trade-off between model complexity and localization performance, we set the encoder depth to 2, which achieved an MAE of 1.03 m on the validation set and 1.55 m on the test set. Moreover, even without data augmentation, our model still outperforms ANVIL and VITAL by over 8%. These experimental results confirm the effectiveness and robustness of the proposed localization model when utilizing SS-RSRP sequences for positioning.

### 4.5. Complexity Analysis

In the fingerprinting-based indoor positioning system, the complexity of the offline construction phase would not affect the real-time performance of the online matching phase. Thus, the time complexity of the offline phase, which mainly comprises TDA and the process of the localization model, is analyzed in detail.

The time complexity of the CWGAN network is expressed as follows:

(21) $$T_{CWGAN}\approx O\left(E\cdot N\cdot \left(L_{G}\cdot h_{G}+L_{D}\cdot h_{D}\right)\right)$$

where *E* is the number of epochs, *N* is the number of training samples, $L_{G}$ and $L_{D}$ are the numbers of layers in the generator and discriminator, and $h_{G}$ and $h_{D}$ are the hidden units. This complexity is comparable to that of a GAN. The only additional computational cost arises from the path generation algorithm. Given *G* generated trajectories, the complexity is O(G), which is negligible compared with the training complexity of the GAN network.

The computational complexity of the proposed localization model is dominated by the attention mechanism and is comparable to that of prior attention-based approaches, including ANVIL and VITAL. Since the sequence length (*L*) is much smaller than the embedding dimension in multi-head attention, the complexity can be expressed as follows:

(22) $$T_{MA}\approx O\left(H\cdot L\cdot {d_{k}}^{2}\right)$$

where *H* represents the number of attention heads and $d_{k}$ is the dimension of keys/queries per head.

Table 9 provides a detailed comparison of the computational complexities of different models, including the total training time and the inference time for a single test sample. Here, GFLOPs denote the giga floating point operations required by the localization model. For traditional machine learning algorithms, the complexity is negligible, since they do not involve explicit model training. During the offline phase, data augmentation (if applicable) and model training are only performed once. The reported training time includes both data augmentation and the training of the localization model.

In terms of GFLOPs, the proposed model is less complex than other attention-based methods such as VITAL and ANVIL. This advantage stems from the fact that VITAL introduces additional computational overhead through patch embedding, while our model adopts a more lightweight multi-head attention structure compared to ANVIL. Regarding training time, methods with data augmentation incur additional costs, since both the augmentation process and the training of the localization model must be considered. However, unlike the complex kernel function computation of Gaussian process regression applied in MapLoc, CWGAN generates new samples with only a single forward propagation. This makes our model more computationally efficient while also producing diverse samples beyond simple interpolation, offering greater feasibility in real-world deployment. Other trajectory-based methods (e.g., WiFiNet and LSTM) achieve shorter training times without data augmentation but fail to capture sufficient sample diversity and, thus, show limited accuracy on small datasets. Since model training occurs only offline, training time does not affect online efficiency. Our method achieves online inferring performance with a latency of about 2 ms. Overall, the computational overhead of our method remains acceptable and constitutes a reasonable trade-off for enhanced localization performance.

## 5. Conclusions and Future Work

In this work, we proposed an attention-based indoor localization system leveraging an extended trajectory map constructed via a novel trajectory-based data augmentation method. By employing a conditional Wasserstein generative network, synthetic fingerprints were generated at unmeasured locations, and a path generation algorithm enriched trajectory diversity. To improve trajectory prediction, a multi-head attention model with a direction-constrained auxiliary loss effectively captured spatial–temporal dependencies in SS-RSS sequences. Extensive experiments in a real 5G indoor environment demonstrate that the proposed system significantly outperforms existing methods, achieving at least a 34% improvement in localization accuracy. The findings confirm that integrating trajectory-based data augmentation with attention modeling enhances robustness and accuracy for positioning in next-generation wireless networks.

As part of future work, we will extend our study to large-scale indoor scenarios where environmental dynamics—such as furniture rearrangement, pedestrian density variations, and layout changes—may lead to distribution shifts and performance degradation. To enhance robustness under such conditions, we plan to investigate data filtering and noise suppression techniques, as well as domain adaptation and transfer learning methods, to improve the generalization capability of the proposed system across diverse environments.

## Figures and Tables

**Table 1:** A comparison of RSS-based related works.

Method	Model Description	Sequence Data	Data Augentation	Extended Trajectory Map	Localization Type
KNN [13]	Weighted k-nearest neighbors	N	N	N	Static
SVM [14]	Support vector machine	N	N	N	Static
RF [15]	Random forest classifier	N	N	N	Static
CNNs [26,27]	Custom and classical CNNs for classification	Y	N	N	Static/Dynamic
CNN-LSTM [35]	A hybrid model of a 1D CNN and LSTM	N	N	N	Static
LSTM [18]	LSTMs using RSS sequences for dynamic localization	Y	N	N	Dynamic
VITAL [22]	Vision transformer neural networks for indoor localization	Y	Y	N	Static
GAN [33]	Generative adversarial networks for data augmentation and DNNs for localization	N	Y	N	Static
ANVIL [23]	An attention neural network with data augmentation on existing points	Y	Y	N	Static
MapLoc [34]	An LSTM localization network with data augmentation at unmeasured locations	Y	Y	N	Dynamic
Proposed	A multi-head attention network with extended trajectory map construction	Y	Y	Y	Dynamic

Note: Y = feature is considered; N = feature is not considered.

**Table 2:** Key parameter settings of the CWGAN.

Parameter	Value
Layers of generator	FC1: (50) × 1
	FC2: (50) × 1
	FC3: (30) × 1
Number of encoder layers ($N_{block}$)	5
Layers of discriminator	(50) × 2
Learning rate of generator	0.001
Learning rate of discriminator	0.0005
Latent dim of noise (*z*)	40
Batch size	128
Optimizer	Adam
Tuning factor (λ)	5
$n_{critic}$	3

**Table 3:** Key parameter settings of the localization model.

Parameter	Value
Length of sequence	4
Batch size	128
Hidden dim of FC layers	128
Number of encoder layers	2
Number of attention heads	4
Encoder layer dim ($d_{model}$)	64
Dropout rate	0.3
Weighting factor (α)	0.1
Angle threshold $\left(\theta_{thr}\right)$	$90^{\circ }$

**Table 4:** Experimental settings.

Parameter	Value
Size of the hall	500 m^2^
Localization area	10 × 7 m
RPs	2112 points
BSs	6 BSs located on three floors
Training dataset	1676 sequences
Validation dataset	420 sequences
Testing dataset	300 sequences
Length of sequence	4
Robot hardware	A 16-line LiDAR sensor
Robot software	Ubuntu 22.04
Mobile device	Huawei P40

**Table 5:** Comparison of localization accuracy across different methods.

Method	MAE (m)	CDF80% (m)	CDF90% (m)	Improvement (%)
KNN	2.34	3.39	4.27	53.42
SVM	3.00	4.11	4.87	63.67
RF	2.06	3.08	3.73	47.09
WiFiNet	1.84	2.73	3.60	40.76
CNN-LSTM	2.30	3.40	3.98	52.61
LSTM	1.71	2.46	3.54	36.26
VITAL	1.70	2.32	3.13	35.88
GAN	2.17	3.24	3.97	49.77
ANVIL	1.67	2.43	3.42	34.73
MapLoc	2.00	2.87	4.27	45.50
Proposed	1.09	1.50	2.23	\

**Table 6:** Localization errors for different sequence lengths.

L	MAE (m)	CDF80% (m)	CDF90% (m)
3	1.68	2.48	3.50
4	1.55	2.23	3.11
5	1.70	2.43	3.44
6	1.82	2.70	3.41
7	2.00	3.00	3.82

**Table 7:** MAE for different numbers of attention heads.

Number of Attention Heads	Training Set (m)	Validation Set (m)	Test Set (m)
1	0.61	1.22	1.65
2	0.61	1.18	1.72
4	0.47	1.08	1.64
8	0.59	1.18	1.67

**Table 8:** MAE for different numbers of encoder layers.

Number of Encoder Layers	Training Set (m)	Validation Set (m)	Test Set (m)
1	0.51	1.2	1.68
2	0.47	1.03	1.55
3	0.53	0.92	1.64
4	0.69	1.24	1.72

**Table 9:** Complexity analysis of different methods.

Method	GFLOPs	Training Time (s)	Responding Time (ms)
KNN	-	-	0.98
SVM	-	-	1.01
RF	-	-	1.05
WiFiNet	0.00319	105	1.74
CNN-LSTM	0.00168	87	2.26
LSTM	0.00243	94	2.01
VITAL	0.00469	92	1.87
GAN	0.00102	312	1.82
ANVIL	0.00371	200	2.07
MapLoc	0.00165	1021	2.14
Proposed	0.00358	427	2.01

## Data Availability

The data that support the findings of this study are available from the corresponding author upon reasonable request.
